# Fungus Causing White-Nose Syndrome in Bats Accumulates Genetic Variability in North America with No Sign of Recombination

**DOI:** 10.1128/mSphereDirect.00271-17

**Published:** 2017-07-12

**Authors:** Jigar Trivedi, Josianne Lachapelle, Karen J. Vanderwolf, Vikram Misra, Craig K. R. Willis, John M. Ratcliffe, Rob W. Ness, James B. Anderson, Linda M. Kohn

**Affiliations:** a Department of Biology, University of Toronto, Mississauga, Ontario, Canada; b New Brunswick Museum, Saint John, New Brunswick, Canada; c Western College of Veterinary Medicine, University of Saskatchewan, Saskatoon, Saskatchewan, Canada; d Department of Biology and Centre for Forest Interdisciplinary Research (C-FIR), University of Winnipeg, Winnipeg, Manitoba, Canada; Carnegie Mellon University; University of Michigan; University of California, Riverside; University of Western Ontario

**Keywords:** clonal reproduction, epidemic, fungal pathogens, population biology, population genomics, spontaneous mutations

## Abstract

Emerging fungal diseases of wildlife are on the rise worldwide, and the white-nose syndrome (WNS) epidemic in North American bats is a catastrophic example. The causal agent of WNS is a single clone of the fungus *Pseudogymnoascus destructans*. Early evolutionary change in this clonal population has major implications for disease ecology and conservation. Accumulation of variation in the fungus through mutation, and shuffling of variation through recombination, could affect the virulence and transmissibility of the fungus and the durability of what appears to be resistance arising in some bat populations. Our genome-wide analysis shows that the clonal population of *P. destructans* has expanded in size from a single genotype, has begun to accumulate variation through mutation, and presents no evidence as yet of genetic exchange among individuals.

**IMPORTANCE** Since its discovery in 2006, the emerging infectious disease known as white-nose syndrome has killed millions of bats in North America, making it one of the most devastating wildlife epidemics in recorded history. We demonstrate that there has been as yet only spontaneous mutation across the North American population of *P. destructans*, and we find no indication of recombination. Thus, selective forces, which might otherwise impact pathogenic virulence, have so far had essentially no genetic variation on which to act. Our study confirmed the time of origin for the first and, thus far, only introduction of *P. destructans* to North America. This system provides an unprecedented opportunity to follow the evolution of a host-pathogen interaction unfolding in real time.

## Abstract (precis)

Since its discovery in 2006, the emerging infectious disease known as white-nose syndrome has killed millions of bats in North America, making it one of the most devastating wildlife epidemics in recorded history. We demonstrate that there has been as yet only spontaneous mutation across the North American population of *P. destructans*, and we find no indication of recombination. Thus, selective forces, which might otherwise impact pathogenic virulence, have so far had essentially no genetic variation on which to act. Our study confirmed the time of origin for the first and, thus far, only introduction of *P. destructans* to North America. This system provides an unprecedented opportunity to follow the evolution of a host-pathogen interaction unfolding in real time.

## INTRODUCTION

White-nose syndrome (WNS) is associated with widespread bat mortality and has driven one of the most common North American bat species, Myotis lucifugus, to the brink of local extinction in eastern North America (1). WNS was first reported in bats at a single location in the New York state in 2006 (2) and has since spread throughout most of eastern North America and recently to a single outlier location in Washington state (3). Challenge experiments have confirmed that *P. destructans* is the causal agent of WNS via Koch’s postulates (4). The fungus is cave-adapted and cold-tolerant and causes cutaneous infection during hibernation, leading to disruption in bats’ torpor-arousal cycles and, ultimately, to the depletion of fat reserves crucial to winter survival (5, 6). European isolates are infectious and cause cutaneous lesions in captive North American and European bats, but mass mortality of bats has not been observed in Europe; host and pathogen apparently are able to coexist there (6–8). This is consistent with the hypothesis that the hardest-hit North American bat species were naive hosts with little intrinsic resistance to *P. destructans*. Coevolution of the partners in this pathosystem might commence with the further accumulation of virulence determinants in the fungus and resistance determinants in the bats.

Evidence from DNA fingerprinting (9) and multilocus sequence typing (MLST) (10, 11) is consistent with the hypothesis that introduction of *P. destructans* to North America was by a single genotype of one mating type. However, those earlier studies did not preclude the possibility that the introduced haplotype harbored some genetic diversity. The North American haplotype closely matches that of an isolate from Europe, where strains of the fungus are genetically diverse and both mating types are found (12).

Our primary question was whether or not the clonal population of *P. destructans* in North America has accumulated substantial genetic variability through mutation. Secondarily, we asked whether or not there is evidence of recombination in *P. destructans*. Previous analyses could not address these issues because only a small proportion of genome-wide variation was sampled. We therefore sequenced the genomes of 17 North American strains of *P. destructans* and combined these sequences with five publicly available genome sequences (see Table S1 in the supplemental material). Collectively, our samples span the current spread of the WNS epidemic across North America. We also sequenced the European strain that has the same haplotype as the North American isolates (3). We aligned the individual genomic reads (Table S1) to the *P. destructans* reference genome (NCBI BioProject PRJNA39257) and identified all high-quality variants in the genome (see the supplemental material and Materials and Methods). Our genome-wide analysis recovered extraordinarily few mutations within the 22 complete genomes of *P. destructans*, showing that the spread of WNS across North America is caused by near-isogenic derivatives of the individual originally introduced to North America.

10.1128/mSphereDirect.00271-17.1 TABLE S1 Strains of *P. destructans* used in this study. Download TABLE S1, PDF file, 0.01 MB. © Crown copyright 2017. 2017 Crown This content is distributed under the terms of the Creative Commons Attribution 4.0 International license.

## RESULTS AND DISCUSSION

**FIG 1:**
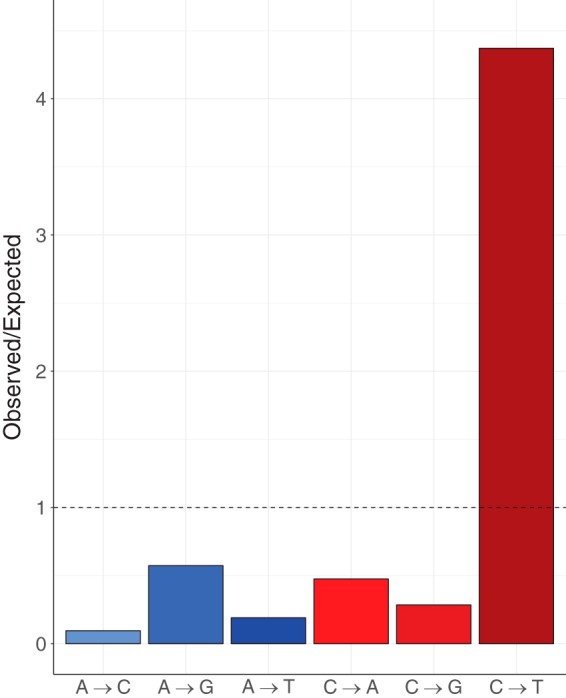
Base spectrum of mutation (Table S1). The excess of C-to-T mutations is consistent with a young population in which natural selection has not begun to act. Cytosine is the most mutable base, and the excess is produced by the tendency of cytosine (and its 5-methyl derivative) to deaminate and then pair with adenine rather than guanine during DNA replication.

All of the confirmed variants (see Data Set S1 in the supplemental material) were nuclear, and none were detected in the 32-kbp mitochondrial genome; the mitochondrial DNA (mtDNA) of the European strain differed by 14 mutations from the North American strain. Our data support five lines of evidence that fit expectations for a young and expanding clonal population (Data Set S1). (i) Variation is exceedingly rare; only 70 variants (7 indels and 63 single nucleotide polymorphisms [SNPs]) were discovered across the 31-Mbp genome among the 22 North American strains. This represents approximately 225×-lower variation than we found between the North American reference genome and the closely related European strain of the same MLST haplotype. (ii) Nearly all variants (61 of 70) were found only once among the 22 North American strains. Such an excess of rare variants suggests that these mutations are new and have not had sufficient time to spread in the population. Consistent with this pattern, Tajima’s D (13), which measures deviations from a stable equilibrium population, is strongly negative (−2.4) and suggestive of a population expansion. (iii) The base spectrum of SNPs resembles that of *de novo* mutation (Fig. 1). If mutations are young, there will not have been time for them to be influenced by natural selection and they should therefore reflect the spectrum of spontaneous mutation. Specifically, we found an excess of C/G-to-T/A transitions (46 of 63 SNPs), which is consistent with the *de novo* mutation spectrum of other fungi, plants, and animals. (iv) Recombination is not detected—for any pair of biallelic loci, the only ways to generate all 4 combinations of alleles are recombination and recurrent mutation (14). Comparing all pairs of SNPs where the minor allele is in at least 2 individuals, we find no evidence of recombination. (v) While the European strain carried the ancestral allele at each of the 70 variant sites identified among the North American strains, it was by far the most divergent from the others, with 15,793 variants at other positions scattered throughout the genome. The European strain is therefore substantially different from the strain that originally founded the North American population, a difference not evident in the previous MLST data (10).

10.1128/mSphereDirect.00271-17.2 DATA SET S1 SNP data. Download DATA SET S1, XLSX file, 0.1 MB. © Crown copyright 2017. 2017 Crown This content is distributed under the terms of the Creative Commons Attribution 4.0 International license.

**FIG 2:**
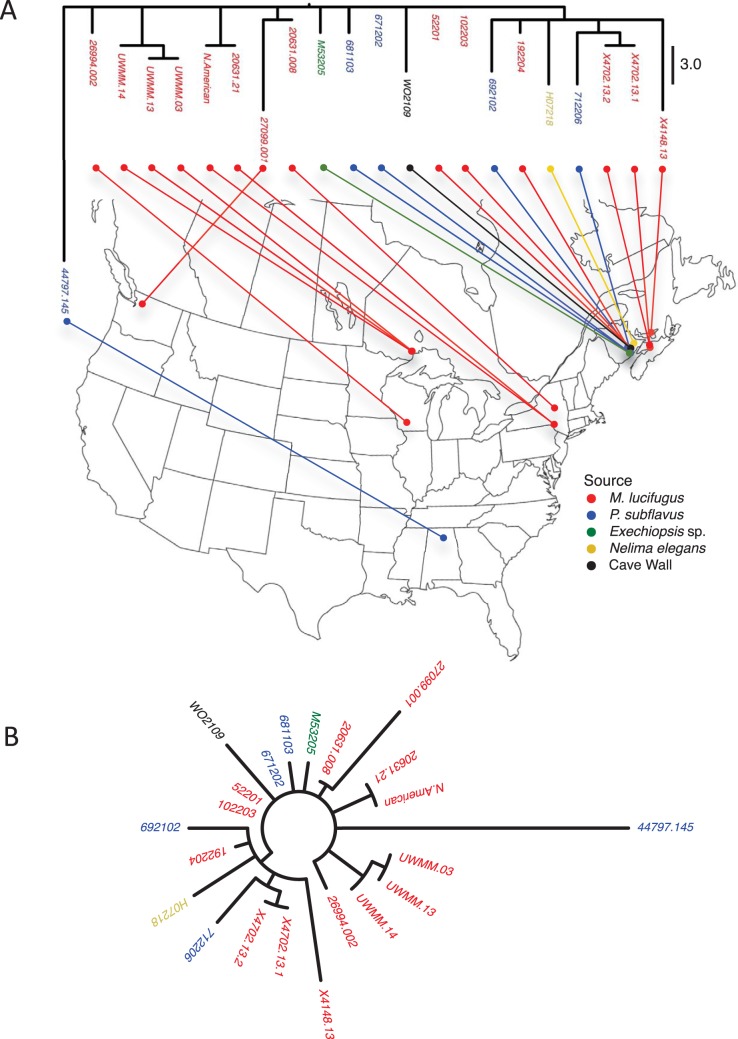
Single-most-parsimonious tree of minimum possible length. (A) Rectangular tree with strain designations and connections to geographic origin. The root of the tree is in the vicinity of the North American/20631.21 genotype, but the exact location is unknown. Note that the North American genotype and the 20631.21 genotype are subcultures of the same strain and were resequenced independently. (B) Unrooted tree showing a starburst pattern of diversification.

The well-supported mutations provided the opportunity to reconstruct the evolutionary relationships of the derivative strains in North America. In parsimony analysis of the 70 variant sites, a single, minimum-length tree (70 steps) of the 23 strains was identified (Fig. 2). As would be expected without recombination, the tree has no internal conflict (consistency index value, 1.0) and the branches therefore represent mutations that occurred only once in the tree. The tree illustrates how the mutant alleles, in addition to being rare, are mostly locally distributed. All of the terminal branches leading to strains represent only singleton mutations, which by definition occur in only one place and are not shared among strains. Also, the strains within the two largest internal clades defined by nonsingleton alleles (represented by internal branches) were geographically restricted to western Ontario and the Maritime Provinces of Canada, respectively, suggesting a local structure. A third internal clade included the genotype that recently appeared in the outlier location in Washington state and the strain from New York state in 2008.

These results address two issues of critical conservation importance. First, North American WNS is caused by a single clone of *P. destructans* that has begun to accumulate new alleles through mutation and is in an early stage of diversification. Second, the fungal population has not yet undergone genetic exchange and recombination. Recombination is always a possibility, even in the absence of two mating types and a sexual cycle, because of the well-known capacity of fungi for parasexuality (15). Given the extreme uniformity of the North American population, individuals of the clone can presumably undergo hyphal anastomosis without triggering a somatic incompatibility response (16); nuclei within the same cytoplasm may then fuse and give rise to recombinant daughter nuclei. With the accumulation of additional variability in the future, the probability of detecting recombination, if it exists, should increase.

Our results raise the issue of how further accumulation of variability will impact the evolution of virulence in the fungus and, in turn, of resistance of host bats. The answer here will depend not only on the ongoing population dynamics of the fungus but also on those of the bats. Evidence suggests that at least one common North American bat species (Eptesicus fuscus) is resistant to or tolerant of infection and has apparently not suffered mass mortality (17). Impacts vary widely for populations of other infected species (1). Moreover, detection of a slight rebound in the M. lucifugus populations hit first by WNS suggests the possibility of some level of increasing resistance in some North American bat populations (18, 19). Of particular concern is that new introductions of *P. destructans* may yet add variability to the North American population of the pathogen and increase the potential for recombination by enabling sexual recombination between mating types; sexual recombination would presumably proceed at a much higher rate than parasexual recombination. Such changes in the fungus population could affect the durability and strength of newly appearing resistance in bats and might even lead to a renewed or expanded epidemic. This system provides an unprecedented opportunity to follow the evolution of a host-pathogen interaction unfolding in real time and highlights the importance of ensuring high levels of biosecurity for invasive pathogens even after they have already been introduced.

## MATERIALS AND METHODS

### *P. destructans* cultivation and DNA isolation.

*P. destructans* was cultivated on complete yeast medium (CYM; 2 g yeast extract, 2 g peptone, 20 g dextrose, 0.5 g magnesium sulfate, 0.46 g monobasic phosphate buffer, and 1 g dibasic phosphate buffer per liter) at 10 C. For DNA isolation, strains were allowed to grow for 4 weeks on sterile cellophane membranes placed on the surface of CYM agar at 10 C. A single colony of each strain was frozen in liquid nitrogen and then lyophilized. Care was taken to ensure that the frozen mycelia did not thaw before complete lyophilization. DNA was isolated by the protocol of Palmer et al. (12). Higher yields of DNA were obtained when the incubation of the mycelia in the lysis buffer was increased to 3 h from 1 h. The crude DNA preparation was purified with a Qiagen Gentra Puregene kit.

### Molecular identification of *P. destructans*.

The identity of the fungus was confirmed through internal transcribed spacer (ITS) gene sequencing. The PCR setup consisted of 12.5 μl of 2× master mix (GoTaq green master mix; Promega, Madison, WI), 2.5 μl each of 5 µM stocks of the ITS1 and ITS4 primers, 6.5 μl of sterile water, and 1 μl of the template DNA (original extract diluted 100-fold). PCR conditions were initial denaturation at 95 C for 2 min and 35 cycles at 95 C for 30 s, 53 C for 30 s, and 72 C for 1 min followed by a final extension at 72 C for 10 min. Agarose gel electrophoresis confirmed amplification along with a 100-bp molecular marker on a 2% agarose gel in Tris-acetate-EDTA (TAE) buffer stained with Sybr Safe (Thermo Fisher). PCR products were purified by precipitation with polyethylene glycol (PEG) before submission for Sanger sequencing at the sequencing facility of The Centre for Applied Genomics (TCAG), Sick Children’s Hospital (Toronto, Ontario, Canada).

### Genome sequencing.

The genomic DNAs of *P. destructans* were sent to TCAG for whole-genome sequencing on the Illumina HiSeq platform. After the samples passed the standard quality control tests (Qubit DNA quantification) for Illumina sequencing, libraries were prepared by the TruSeq Nano protocol and paired-end reads of 151 bp were obtained (Illumina, San Diego, CA). An average of ca. 35 M reads were obtained from each strain for an average coverage depth of 160×. The sequenced strains included the European and North American reference strains (see Table S1 in the supplemental material) as a two-point reference for the SNP discovery process for the other North American strains.

### European origin of North American *P. destructans*.

A recent MLST study (10) used eight polymorphic gene loci to identify eight haplotypes in Europe, of which one, haplotype 1, was identified as the source of *P. destructans* in North America. The eight sequences of haplotype 1 were therefore concatenated and used as a reference for alignment of the Illumina reads of each of the strains in our sample with Geneious 9.1. The genome sequences of all strains in our sample were identical to the haplotype 1 sequences—no variation was detected in these regions among the 22 North American strains.

### Data preprocessing and read alignment.

Raw sequence data (Fastq) from each strain were initially preprocessed with the Trimmomatic tool to remove the custom next-generation sequencing adapters/primers from the raw data. The preprocessed reads were aligned to the *P. destructans* reference genome (*Geomyces destructans* Sequencing Project, Broad Institute of Harvard and MIT; NCBI BioProject PRJNA39257) by using the Burrows-Wheeler Aligner tool (20). Overall, around 99.9% of the reads aligned to the *P. destructans* reference genome. A binary version (BAM format) of the SAM file was created by using the SortSam command. These files were readied for variant discovery by using Markduplicates and BuildBamIndex functions. These preprocessing functions were part of the Picard tools package (http://picard.sourceforge.net). To avoid alignment artifacts due to indels and to improve SNP detection, IndelRealigner and RealignerTargetCreator from GATK were used (21–23). These functions perform a local realignment of indels, resulting in a decrease in the number of false-positive SNP calls.

### Variant calling and filtering.

We used the GATK tool “UnifiedGenotyper” (v3.5) (22) to identify SNPs and short indels. All 23 strains were run jointly, with the ploidy value set to 2. Given that *P. destructans* is haploid, any site identified as a heterozygote site indicates potential mapping errors from paralogous loci or DNA sample contamination. In identifying extremely rare variants, mapping errors are a common cause of false positives, and filtering sites with two haplotypes has proven effective in previous work (24, 25). We applied the following filters for candidate variants: (i) the genotype quality (“GQ”) value was greater than 10; (ii) all called individuals were homozygous; (iii) only two alleles were present at the site. All filtered candidate variants were visually inspected in the Integrative Genomics Viewer (IGV) (26) to further eliminate the possibility of erroneous calls. We excluded all variants from the European strain from the analysis.

### Maximum parsimony analysis.

For parsimony analysis, the 37 strains were considered taxa, the 83 variant sites were considered characters, and their alleles were considered character states. The initial analysis was done by hand; a single-most-parsimonious tree of the shortest possible length (83 steps) and no internal conflict was identified. PAUP4.0 was used to confirm the optimal tree and to generate Fig. 1.

### Accession number(s).

Accession numbers for the *P. destructans* strains used in this study are presented in Table S1.

## ADDENDUM IN PROOF

During the course of this study, 19 additional North American strains were analyzed (J. Trivedi, K. Vanderwolf, V. Misra, C. K. R. Willis, J. Ratcliffe, J. B. Anderson, and L. M. Kohn, bioRxiv, 2017, https://doi.org/10.1101/121038). These 19 are not included in this study because their data accession numbers were not available to us.
