# Supplementary material for: Fungus Causing White-Nose Syndrome in Bats Accumulates Genetic Variability in North America with No Sign of Recombination
Source: mSphere. 2017 Jul 12;2(4):e00271-17. doi: 10.1128/mSphereDirect.00271-17 (PMC5506559; doi:10.1128/mSphereDirect.00271-17)
Supplement: TABLE S1 [file sph004172318st1.pdf]

Table S1. Strains of *P. destructans* used in this study.

Strain designation	Substrate	Location[b]	Provider[c]	Year	Sequence accession number
102203	*Myotis lucifugus*	White Cave, NB	K. Vanderwolf	2012	SRR5755627
192204	*M. lucifugus*	Markhamville Mine, NB	K. Vanderwolf	2012	SRR5755628
52201	*M. lucifugus*	White Cave, NB	K. Vanderwolf	2012	SRR5757331
671202	*Perimyotis subflavu*s	Glebe Mine, NB	K. Vanderwolf	2013	SRR5755625
681103	*P. subflavu*s	Glebe Mine, NB	K. Vanderwolf	2013	SRR5755626
692102	*P. subflavu*s	Markhamville Mine, NB	K. Vanderwolf	2013	SRR5755631
712206	*P. subflavu*s	Markhamville Mine, NB	K. Vanderwolf	2013	SRR5755632
European	*M. myotis*	Thuringa, Germany	V. Misra	2009	SRR5755629
H07218	*Nelima elegans*	Dorchester Mine, NB	K. Vanderwolf	2013	SRR5755630
M53205	*Exechiopsis sp.*	Glebe Mine, NB	K. Vanderwolf	2013	SRR5755633
N. American[a]	*M. lucifugus*	Williams Hotel, NY	V. Mishra	2008	SRR5755634
X4702.13.1	*M. lucifugus*	NS	K. Vanderwolf	2013	SRR5755620
X4702.13.2	*M. lucifugus*	NS	K. Vanderwolf	2013	SRR5755621
UWMM.03	*M. lucifugus*	Thunder Bay, ON	C. Willis	2015	SRR5755618
UWMM.13	*M. lucifugus*	Thunder Bay, ON	C.Willis	2015	SRR5755619
UWMM.14	*M. lucifugus*	Thunder Bay, ON	C. Willis	2015	SRR5755623
WO2109	Cave Wall	White Cave, NB	K. Vanderwolf	2015	SRR5755624
X4148.13	*M. lucifugus*	PEI	K. Vanderwolf	2013	SRR5755622
27099-001	*M. lucifugus*	King County, WA	n/a	2016	SRR3545533
44797-145	*P. subflavu*s	Jackson County, Al	n/a	2015	SRR3545532
26994-002	*M. lucifugus*	Iowa County, WI	n/a	2016	SRR3545531
20631-008	*M. lucifugus*	Albany County, NY	n/a	2008	SRR3545530
20631-21[a]	*M. lucifugus*	Williams Hotel, NY	n/a	2008	SRR1952982

[a]These represent different subcultures of the same strain sequenced by the Broad Institute to generate the reference assembly used here. We retained these two as different strains because they were re-sequenced independently from different subcultures. That they have the same genotype with respect to the 70 variant positions constitutes an internal control; there were no new mutations during independent culture in different laboratories.
[b]Two-letter designation for states and provinces (except PEI).
[c]Note: Substrate name was specified by the collector or the accession record.
